# Connexin43 mutations linked to skin disease have augmented hemichannel activity

**DOI:** 10.1038/s41598-018-37221-2

**Published:** 2019-01-10

**Authors:** Miduturu Srinivas, Thomas F. Jannace, Anthony G. Cocozzelli, Leping Li, Nefeli Slavi, Caterina Sellitto, Thomas W. White

**Affiliations:** 1 Department of Biological and Vision Sciences, State University of New York College of Optometry, New York, NY 10036 USA; 2 Department of Physiology and Biophysics, Stony Brook University, Stony Brook, NY 11794 USA; 3 MS program in Biomedical Sciences (Physiology and Biophysics track), Stony Brook University, Stony Brook, NY 11794 USA

## Abstract

Mutations in the gene (*GJA1*) encoding connexin43 (Cx43) are responsible for several rare genetic disorders, including non-syndromic skin-limited diseases. Here we used two different functional expression systems to characterize three Cx43 mutations linked to palmoplantar keratoderma and congenital alopecia-1, erythrokeratodermia variabilis et progressiva, or inflammatory linear verrucous epidermal nevus. In HeLa cells and *Xenopus* oocytes, we show that Cx43-G8V, Cx43-A44V and Cx43-E227D all formed functional gap junction channels with the same efficiency as wild-type Cx43, with normal voltage gating and a unitary conductance of ~110 pS. In HeLa cells, all three mutations also localized to regions of cell-cell contact and displayed a punctate staining pattern. In addition, we show that Cx43-G8V, Cx43-A44V and Cx43-E227D significantly increase membrane current flow through formation of active hemichannels, a novel activity that was not displayed by wild-type Cx43. The increased membrane current was inhibited by either 2 mM calcium, or 5 µM gadolinium, mediated by hemichannels with a unitary conductance of ~250 pS, and was not due to elevated mutant protein expression. The three Cx43 mutations all showed the same gain of function activity, suggesting that augmented hemichannel activity could play a role in skin-limited diseases caused by human Cx43 mutations.

## Introduction

Connexins (Cx) are a family of proteins that make gap junction channels, and allow the direct passage of small molecules between adjacent cells^1,2^. Connexins oligomerize into hemichannels (also called connexons) that contain six connexin monomers during transit through the ER-Golgi pathway^3,4^. Hemichannels are transported to the plasma membrane, where they can act as functional channels on their own^5–7^, or move to regions of cell contact and dock with a partner hemichannel in an adjacent cell to form a gap junction channel^8^. Gap junction channels formed by different connexins have unique gating, conductance and permeability characteristics^8–13^, and these functional differences are important, since one type of connexin cannot be functionally replaced by a different connexin in genetically engineered mice^14–16^. The activity of hemichannels can change under conditions of stress, allowing the flux of molecules like Ca^2+^, ATP, glutamate, or NAD^+^ across the cell membrane and provoking a variety of physiological responses^17,18^.

Mutations in ten of the human connexin genes have already been linked to twenty-eight distinct genetic diseases^19^. Eleven of these are skin disorders with an overlapping spectrum of phenotypes that are caused by mutations in five of the connexin genes^20^. Mutations in one of these genes, *GJA1* gene encoding Cx43, cause a number of rare genetic diseases, including skin disease^20,21^. The first *GJA1* mutation identified in an isolated epidermal disorder was the Cx43-G8V mutation linked to palmoplantar keratoderma and congenital alopecia-1 (PPKCA1, also called keratoderma-hypotrichosis-leukonychia totalis)^22^. PPKCA1 is a dominant disorder characterized by severe hyperkeratosis, congenital alopecia and leukonychia^23^. Subsequently, the Cx43-A44V and Cx43-E227D mutations were found to cause erythrokeratodermia variabilis et progressiva (EKVP)^24^. EKVP is another dominant disorder, which can be caused by mutations in three different connexin genes^20^. It is characterized by hyperkeratosis that can be widespread over the body, or limited to a small area. About half of the patients with EKVP also have palmoplantar keratoderma^25,26^. In EKVP caused by Cx43 mutations, patients also had prominent white lunulae and periorificial darkening^24^. Finally, the same Cx43-A44V mutation linked to EKVP, was identified in a patient with inflammatory linear verrucous epidermal nevus (ILVEN)^27^. ILVEN is characterized by pruritic, erythematous, hyperkeratotic papules linearly distributed along Blaschko’s lines, and is caused by somatic, rather than germline mutations^28^. The three Cx43 mutations linked so far to human genetic skin disease are all single amino acid substitutions whose functional consequences have not been fully characterized.

Studies of several mutations in other connexins linked to skin disease have suggested a generalized role for altered hemichannel activity in the connexin skin disorders^20,29–32^. Increases in hemichannel activity have been described for Cx26 mutations causing both Keratitis-Ichthyosis-Deafness (KID) syndrome and palmoplantar keratoderma (PPK) with deafness^33–36^. Cx30 mutations associated with hidrotic ectodermal displaysia (HED) elicited large currents and increased ATP leakage in cells, consistent with altered hemichannel function^37^. In a similar fashion, Cx31 mutations causing EKVP were shown to result in increased hemichannel activity, ATP leakage, and necrotic cell death when expressed in transfected cells^38^. Even in the case of Cx43, the initial characterization of the Cx43-G8V mutation linked to PPKCA1 in transfected cells showed increased whole cell membrane current, Ca^2+^ influx, and cell death when compared to wild-type Cx43, which could have been mediated by an increase in the activity of hemichannels^22^.

Here we report the functional characterization of all three of the Cx43 mutations that have been linked to non-syndromic human skin disease thus far, Cx43-G8V, Cx43-A44V and Cx43-E227D. Using cRNA injected *Xenopus* oocytes or transiently transfected HeLa cells, we demonstrate that all three mutations form functional gap junctions as efficiently as wild-type-Cx43, with no obvious differences in voltage gating, unitary conductance, protein expression, or cellular localization. We further observed that Cx43-G8V, Cx43-A44V and Cx43-E227D all formed functional hemichannels with greatly increased membrane current flow, a feature not shared by wild-type Cx43. These results suggest that the augmentation of hemichannel function shared by all three mutations may play a role in the pathophysiology of human Cx43 mutations linked to skin disease.

## Results

### Wild-type and mutant forms of Cx43 show equivalent levels of protein expression in *Xenopus* oocytes

**Figure 1:**
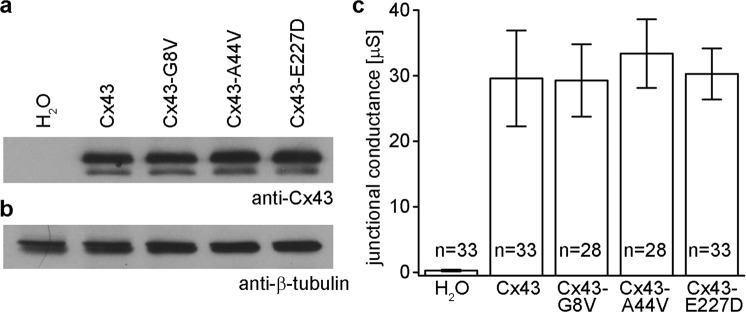
Wild-type and mutant forms of Cx43 are equivalently expressed and form functional gap junctions in paired *Xenopus* oocytes. (**a**) Equal amounts of membrane extracts were first probed with an antibody that recognized Cx43. H_2_O injected controls did not express Cx43 as expected. Wild-type Cx43, Cx43-G8V, Cx43-A44V, and Cx43-E227D were readily detected in lanes corresponding to each injection condition with similar band intensities. (**b**) To confirm equal sample loading, blots were stripped and reprobed with an antibody against β-tubulin, which was present at comparable levels in all lanes. (**c**) Gap junctional conductance levels for cell pairs expressing either wild-type, or mutant, forms of Cx43 were all significantly greater than the H_2_O injected negative controls (p < 0.05, one way ANOVA), but not significantly different from each other. Values are the mean ± SEM.

Equivalent protein expression of wild-type and mutant Cx43 was verified by western blotting of cRNA injected *Xenopus* oocytes. Immunoblotting for Cx43 revealed ~43 kDa bands of similar intensity in lanes corresponding to oocytes injected with either wild-type Cx43, Cx43-G8V, Cx43-A44V, or Cx43-E227D cRNAs. No Cx43 band was detected in H_2_O injected control cells (Fig. 1a). When the blot was re-probed for β-tubulin, it was detected at similar intensity in all samples, confirming equal protein loading (Fig. 1b). These data showed that levels of Cx43 protein synthesis following cRNA injection were similar for mutant and wild-type Cx43.

### Cx43 mutations form functional gap junction channels in *Xenopus* oocytes

To examine the ability of Cx43 skin disease mutations to make gap junction channels, wild-type and mutant Cx43 were expressed in *Xenopus* oocyte pairs and gap junctional conductance was measured (Fig. 1c). Control oocyte pairs injected with water showed negligible conductance (0.28 µS), while cells with wild-type Cx43 channels had an average conductance of 29.6 µS. Cell pairs expressing Cx43-G8V, Cx43-A44V, or Cx43-E227D had mean conductance levels of 29.3, 33.4 or 30.3 µS respectively. Conductance levels of cell pairs expressing either mutant, or wild-type, forms of Cx43 were all significantly greater than the H_2_O injected negative controls (one way ANOVA, p < 0.05), but not significantly different from each other, consistent with the equivalent levels of Cx43 protein expression (Fig. 1a). Thus, all three of the skin disease associated Cx43 mutations formed gap junction channels with macroscopic conductance levels equal to wild-type Cx43.

### Skin disease mutations do not affect Cx43 voltage gating

**Figure 2:**
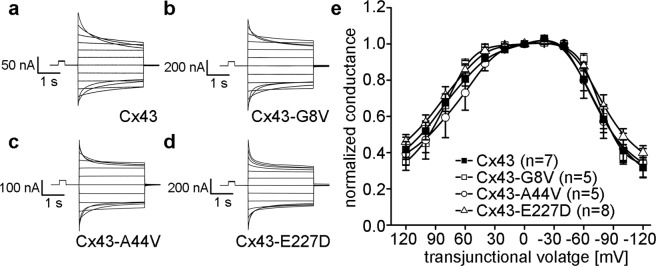
Cx43 mutations do not alter gap junction voltage gating. Oocyte pairs were subjected to hyperpolarizing and depolarizing transjunctional potentials (V_j_) while recording junctional currents (I_j_). (**a**) Wild-type Cx43 gap junction channels had I_j_s that decreased symmetrically at higher values of V_j_. I_j_s between cell pairs expressing Cx43-G8V (**b**), Cx43-A44V (**c**), or Cx43-E227D (**d**) behaved in a similar fashion. (**e**) Steady-state voltage gating of wild-type Cx43 (filled squares) showed an approximately symmetric decline in steady state conductance at increasing values of V_j_. Data from cell pairs expressing Cx43-G8V (open squares), Cx43-A44V (open circles), or Cx43-E227D (open triangles) were similar to wild-type Cx43.

To examine whether skin disease causing mutations altered the voltage gating properties of Cx43 gap junction channels, oocyte pairs were subjected to hyperpolarizing and depolarizing transjunctional potentials (V_j_) while recording junctional currents (I_j_). As previously described^39–41^, I_j_s of wild-type Cx43 gap junction channels decreased symmetrically in a voltage-dependent manner (Fig. 2a). Channels in cell pairs expressing Cx43-G8V, Cx43-A44V, or Cx43-E227D (Fig. 2b–d) behaved in a similar fashion. Steady-state voltage gating was compared by plotting V_j_ (normalized to the value at ±20 mV) against G_j_ (Fig. 2e). Analysis of wild-type Cx43 showed an approximately symmetric decline in steady state conductance at increasing values of V_j_, as has been reported previously^33,40,42^. Cell pairs expressing Cx43-G8V, Cx43-A44V, or Cx43-E227D displayed steady state gating that strongly resembled that of wild-type Cx43.

### Cx43-G8V, Cx43-A44V, and Cx43-E227D all form active hemichannels in unpaired *Xenopus* oocytes

**Figure 3:**
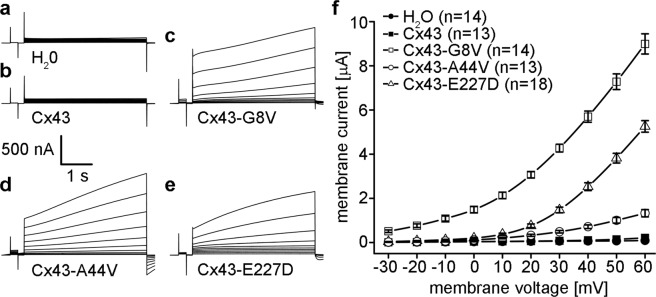
Cx43 mutations induce large hemichannel currents in *Xenopus* oocytes. Single cells were clamped at a holding potential of −40 mV and subjected to voltage pulses ranging from −30 to +60 mV in 10 mV steps. H_2_O (**a**) and wild-type Cx43 (**b**) injected cells displayed negligible membrane currents. Cx43-G8V (**c**), Cx43-A44V (**d**), and Cx43-E227D (**e**) expressing oocytes displayed much larger hemichannel currents than wild-type Cx43. (**f**) Steady-state currents from each pulse were plotted as a function of membrane voltage. Steady state currents in wild-type Cx43 (filled squares) or H_2_O injected control cells (filled circles) were negligible at all tested membrane voltages. Cx43-G8V (open squares) expressing cells exhibited significantly increased steady-state currents at all voltages compared to either control or wild-type Cx43 oocytes. Cx43-A44V (open circles), or Cx43-E227D (open triangles) currents were similar to those observed in control cells at negative voltages, but increased at positive potentials. Data are the mean ± SEM.

To test for changes in hemichannel activity, cRNAs encoding wild-type and mutant forms of Cx43 were injected into single *Xenopus* oocytes that had also received antisense oligonucleotides directed against the endogenous *Xenopus* Cx38^43,44^. Membrane currents were recorded while the cells were clamped to different membrane voltages. Oocytes injected with H_2_O instead of connexin cRNA showed insignificant current flow at membrane voltages between −30 and +60 mV (Fig. 3a). We found that oocytes expressing wild-type Cx43 also showed negligible membrane current flow between −30 and +60 mV (Fig. 3b). In sharp contrast, *Xenopus* oocytes expressing Cx43-G8V, Cx43-A44V, or Cx43-E227D all displayed large outward hemichannel currents upon depolarization (Fig. 3c–e).

To compare differences in hemichannel activity, mean currents were plotted against the membrane potential (Fig. 3f). H_2_O injected control cells, or wild-type Cx43 expressing cells showed minimal membrane currents at all tested voltages. The Cx43-G8V injected cells had much larger hemichannel currents than wild-type Cx43 injected cells at all tested membrane potentials. Cx43-A44V injected oocytes displayed membrane currents that were greater than wild-type Cx43 at membrane potentials ≥+20 mV. Cx43-E227D injected cells had larger membrane currents than wild-type Cx43 at membrane potentials ≥+10 mV. This increased membrane current implied the presence of augmented hemichannel activity by the Cx43 mutants associated with genetic skin disease.

**Figure 4:**
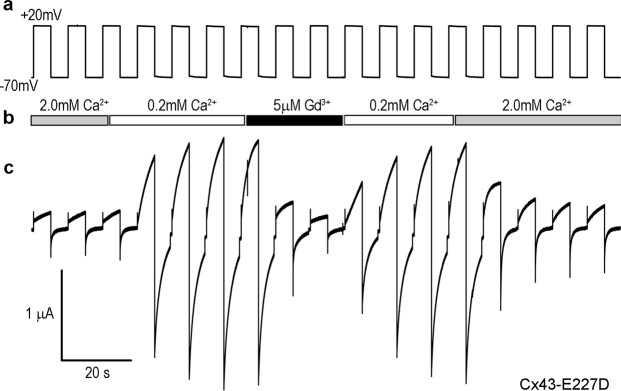
Calcium and gadolinium ions block mutant Cx43 hemichannel activity. Oocytes expressing Cx43 mutations were sequentially stepped from −70 mV to +20 mV (**a**) while being perfused with medium containing 2 mM Ca^2+^, 0.2 mM Ca^2+^, or 5 µM Gd^3+^ (**b**). In the example shown for a Cx43-E227D expressing cell (**c**), hemichannel currents were initially suppressed by 2 mM Ca^2+^, and increased markedly when the extracellular calcium was reduced to 0.2 mM. Hemichannel currents were also suppressed by perfusion with 5 µM Gd^3+^, and recovered rapidly when Gd^3+^ was washed out with 0.2 mM Ca^2+^. Currents were suppressed again by increasing the extracellular calcium to 2.0 mM.

Connexin hemichannels are known to be blocked by the presence of extracellular cations such as calcium and gadolinium^6,45,46^. To confirm that currents observed in single cells expressing the Cx43 mutations were mediated by connexin hemichannels, oocytes were successively stepped from −70 mV to +20 mV while being perfused with extracellular medium containing either 2 mM Ca^2+^, 0.2 mM Ca^2+^, or 5 µM Gd^3+^. In an example shown for a Cx43-E227D expressing oocyte (Fig. 4), hemichannel currents were initially suppressed by the presence of 2 mM Ca^2+^, and increased markedly when the extracellular calcium in the perfusate was reduced tenfold to 0.2 mM. Cx43-E227D hemichannel currents were also efficiently suppressed by perfusion with 5 µM Gd^3+^, and recovered rapidly when the Gd^3+^ was washed out with medium containing 0.2 mM Ca^2+^. Finally, Cx43-E227D hemichannel currents were suppressed back to their initial levels by increasing the extracellular calcium back to 2.0 mM. On average, Cx43-E227D hemichannel currents were inhibited 90 ± 4% (mean ± SD, n = 3) following perfusion with 5 µM Gd^3+^. Similar results were obtained for Cx43-G8V (86 ± 9%, n = 3) and Cx43-A44V (87 ± 3%, n = 3). These data suggest that the increased membrane current seen in oocytes expressing mutant forms of Cx43 is due connexin hemichannel activity.

### Single hemichannels formed by skin disease mutations

**Figure 5:**
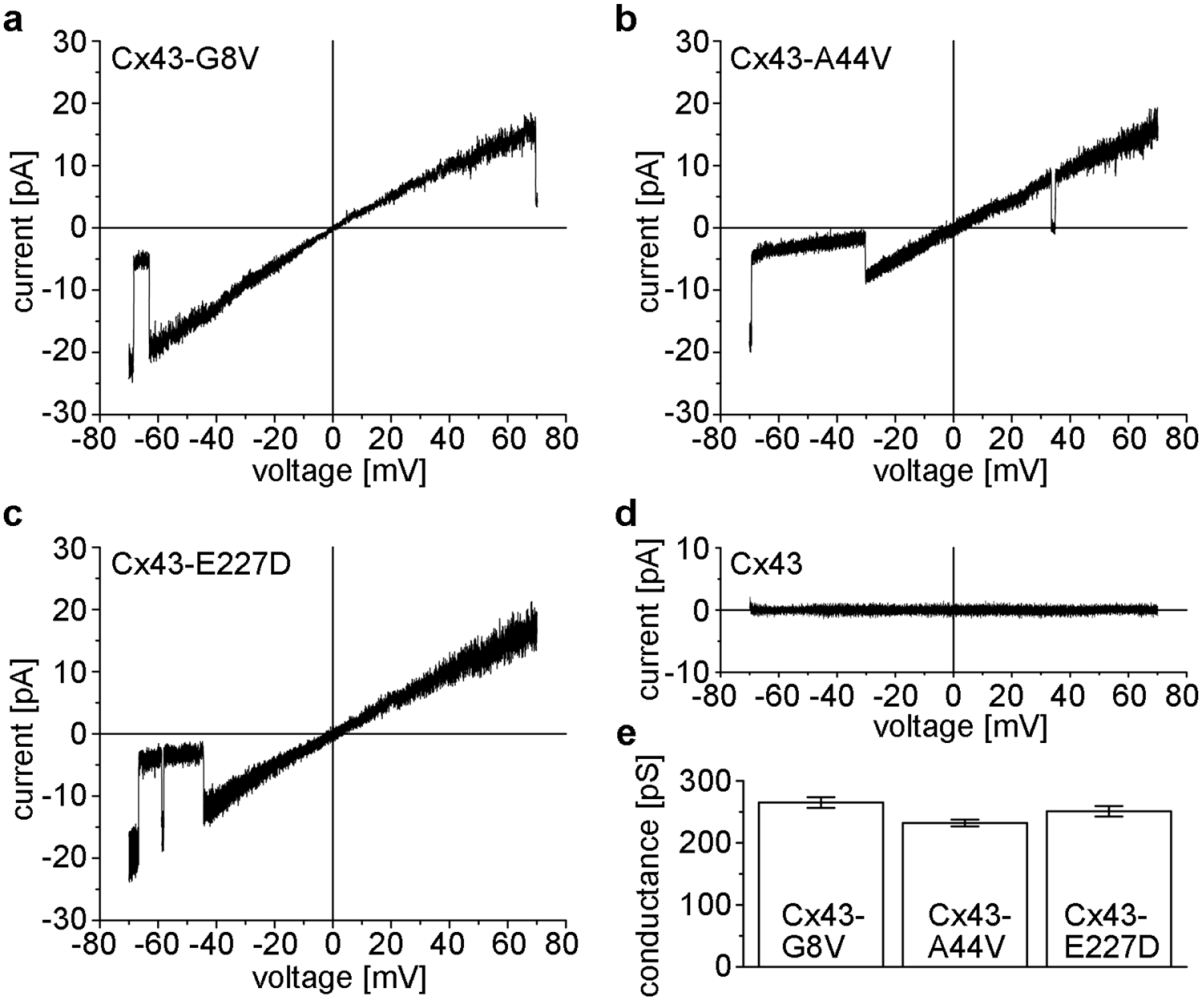
Representative examples of patch clamp recordings from cell-attached patches containing single Cx43-G8V (**a**), Cx43-A44V (**b**), and Cx43-E227D (**c**) hemichannels in symmetric 140 mM KCl solutions. Single hemichannel currents were recorded in response to 8-s voltage ramps between −70 and +70 mV. Current-voltage relations for Cx43-A44V and Cx43-E227D were linear, whereas those of Cx43-G8V showed slight inward rectification. All three mutations show closing transitions to subconductance states. Occasional transitions to the fully closed state are also seen (**b)**. (**d**) Cell-attached patches from wild-type Cx43 injected oocytes failed to show single channel activity. (**e**) Mean slope conductances measured at V_m_ = 0 are similar for the three mutant hemichannels. Values represent the mean ± SEM from 5 to 7 recordings.

The conductance and gating of hemichannels formed by mutant Cx43 subunits was explored using cell-attached or excised patch recordings. *Xenopus* oocytes expressing mutant Cx43 subunits showed large-conductance channels in symmetric 140 mM KCl. Figure 5 shows currents of single Cx43-G8V, Cx43-A44V and Cx43-E227D hemichannels obtained by applying ±70 mV voltage ramps. Open channel currents of Cx43-A44V and Cx43-E227D were largely linear over the voltage range whereas Cx43-G8V hemichannel currents exhibited slight inward rectification. Mean slope conductances, measured at V_m_ = 0 mV, were 265 ± 8.6 for Cx43-G8V (n = 5), 232 ± 5.5 for Cx43-A44V (n = 6), and 251 ± 8.4 (n = 7) for Cx43-E227D. Such large conductance channels were never observed in any of the *Xenopus* oocytes expressing wild-type Cx43.

**Figure 6:**
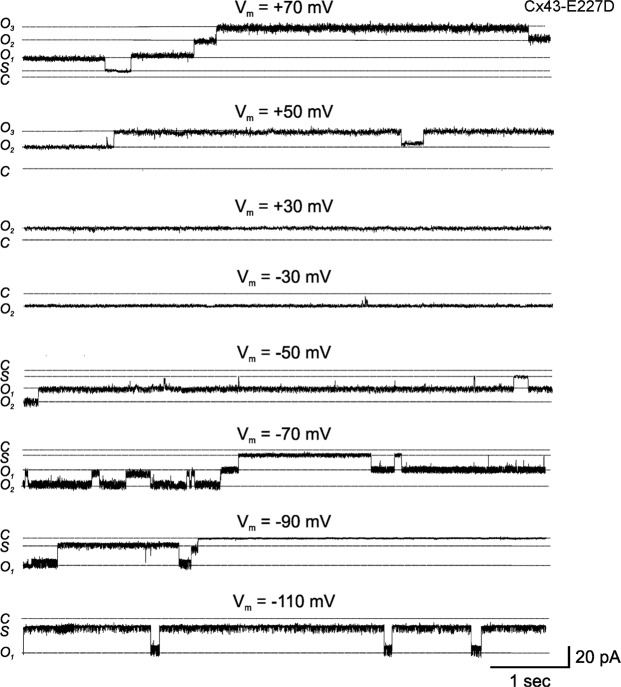
Gating of single Cx43-E227D hemichannels at positive and negative voltages. Representative examples of recordings from a Cx43-E227D expressing oocyte in a cell-attached patch configuration containing one to two open channels at voltages ranging from −110 to +70 mV. Cx43-E227D hemichannels were predominantly open at inside positive voltages. Occasional closures to subconductance states were observed at high positive voltages. In contrast, Cx43-E227D hemichannels showed voltage-dependent closures to negative voltages. While channels are predominantly open at −30 mV, dwell times in the open state decreased with hyperpolarization to −50, −70 and −90 mV. Residence in subconductance states or the fully closed state was favored at the more hyperpolarized voltages. The zero current level, i.e. the fully closed state (C), the current levels of one (O1) or two fully open hemichannels (O2) and that of the subconductance state (S) are depicted by dotted lines. The zero current level was determined as described in Methods.

Our single-channel I–V relationships appeared to indicate that all three mutant Cx43 hemichannels could open at negative voltages. Figure 6 shows current traces from an oocyte expressing Cx43-E227D at different voltages. At positive voltages, Cx43-E227D channels were predominantly open (see traces at V_m_ = +30 mV, +50 mV and +70 mV). Occasional closures to subconductance states at voltages exceeding 50 mV were observed, but residence times in these states did not appear to occur in a voltage dependent manner. At small negative voltages, Cx43-E227D hemichannels showed a high open probability (see traces at V_m_ = −30 mV and −50 mV). With increasingly negative voltages, residence times decreased in the open state, and channels showed frequent closures to subconductance states. Although slow transitions between open and fully closed states were observed (see trace at V_m_ = −90 mV), they were infrequent and appeared to exhibit weak sensitivity to voltage. Similar results were obtained with Cx43-G8V and Cx43-A44V.

### Cx43 mutations also form gap junctions in transfected HeLa cells

**Figure 7:**
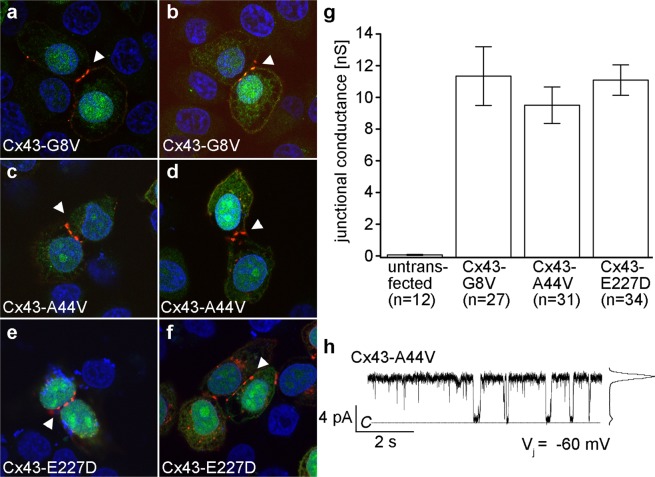
Expression of mutant Cx43 in transfected HeLa cells results in targeting to gap junction plaques and formation of functional intercellular channels. Cx43-G8V (**a,b**), Cx43-A44V (**c,d**), and Cx43-E227D (**e,f**) transfected cells (blue DAPI stain) displayed a strong Cx43 (red) labeling that concentrated at cell-to-cell interfaces (white arrowheads) and correlated with GFP (green) fluorescence. (**g**) Measurement of gap junctional coupling in transfected cell pairs showed that all three Cx43 mutations induced similar high levels of conductance. (**h**) A single gap junction channel recording for Cx43-A44V shows transitions between the fully open state and closed state with a unitary conductance of 113 pS. Data are the mean ± SEM.

To determine mutant protein localization and function in mammalian cells, gap junctional communication-deficient HeLa cells were transiently transfected with the mutant forms of Cx43. Immunofluorescent staining verified protein expression and localization for Cx43-G8V (Fig. 7a,b), Cx43-A44V (Fig. 7c,d), and Cx43-E227D (Fig. 7e,f). All three mutations showed proper trafficking to the cell membrane, especially at the regions of cell-to-cell contact, as shown by punctate staining (white arrowheads). Thus, Cx43 mutant proteins were properly expressed, and junctionally targeted in mammalian cells.

The ability of Cx43 mutants to form functional gap junction channels was also analyzed by dual whole cell patch clamp in the transiently transfected HeLa cells (Fig. 7g). As expected^10,47^, untransfected HeLa cells failed to form gap junctions, with a mean conductance of 0.06 ± 0.03 nS. Consistent with our results obtained in paired *Xenopus* oocytes above (Fig. 1), the mean junctional conductance of Cx43-G8V, Cx43-A44V, and Cx43-E227D cell pairs were 11.3 ± 1.9, 9.5 ± 1.2, and 11.1 ± 1.0 nS, respectively, values that were more than two orders of magnitude greater than the untransfected cell pairs, but not statistically different from each other (p > 0.05, one way ANOVA).

Previous biophysical studies have documented that wild-type Cx43 forms gap junction channels with a unitary conductance of ~110 pS^39,48–50^. To determine if the Cx43-G8V, Cx43-A44V, and Cx43-E227D mutations altered single channel conductance, unitary currents were measured in poorly coupled cell pairs. An example of a single-channel current recorded from a cell pair transfected with Cx43-A44V is shown in Fig. 7h. At a transjunctional voltage of −60 mV, the single-channel was primarily in the open state, with a few transitions to the closed state. An amplitude histogram of the recording, illustrated on the right of the trace, showed a major peak at 6.8 pA, corresponding to the fully open state, and yielding a unitary conductance of 113 pS. The Cx43-G8V, Cx43-A44V, and Cx43-E227D mutations all displayed unitary conductance of gap junction channels similar to wild-type Cx43, with mean values (±SEM) of 110 ± 9 (n = 2), 113 ± 8 (n = 3), and 109 ± 8 pS (n = 3) respectively.

## Discussion

We have functionally characterized three human Cx43 mutations linked to non-syndromic skin-limited genetic disease. Cx43-G8V, Cx43-A44V and Cx43-E227D all formed functional gap junctions as efficiently as wild-type Cx43, with similar voltage gating, unitary conductance, protein expression, and cellular localization. In addition, Cx43-G8V, Cx43-A44V and Cx43-E227D formed functional hemichannels that mediated greatly increased membrane current flow. We found that wild-type Cx43 failed to form ion-conducting hemichannels under physiological conditions, as has been previously reported^46,51–53^. The shared gain of hemichannel function by Cx43-G8V, Cx43-A44V and Cx43-E227D suggests that augmented hemichannel activity could be a common feature of Cx43 mutations linked to skin disease.

In the original clinical reports of these mutations, Cx43-G8V was reported to target to cellular interfaces and support the intercellular passage of fluorescent dye as a GFP tagged construct^22^, consistent with our experimental data. In contrast, Cx43-A44V and Cx43-E227D failed to localize at cell-cell junctions, and aggregated in the Golgi apparatus when transfected into HeLa cells as hemagglutinin-tagged constructs, with no test of channel function in this study^24^. In contrast to this report, we found that untagged versions of both Cx43-A44V and Cx43-E227D targeted to cellular interfaces and supported gap junctional coupling in two different expression systems. In addition, the gap junction channels that we recorded had unitary conductance and voltage gating properties that would be expected for Cx43. We suspect that the hemagglutinin-tag, or possible high levels of overexpression, may have altered the improper localization reported by Boyden *et al*.^24^.

Our single channel studies further revealed that the unitary conductance of hemichannels formed by all three mutants is ~250 pS, close to twice that of their corresponding gap junction channels, as would expected from the series docking of two identical hemichannels in a cell-cell channel^8^. We did not observe single hemichannels in wild-type Cx43 injected oocytes, but our values of unitary conductance for the three different mutants are similar to the ∼220 pS unitary conductance previously reported for wild-type Cx43 hemichannels in HeLa cells^54,55^. In contrast, the gating characteristics of hemichannels formed by Cx43 mutants exhibit significant differences from previous reports of wild-type Cx43 hemichannels^55,56^. In solutions containing Ca^2+^/EGTA (free Ca^2+^ concentrations <10^−7^ M), we found that all three mutant hemichannels primarily resided in the fully open state at low to moderate inside negative voltages. All of the mutant hemichannel currents also exhibited gating to subconductance states at negative voltages, whereas depolarizing voltages produced only brief transitions to intermediate states. These hemichannel properties are markedly different from those of wild-type Cx43, which required depolarization exceeding +40 mV for their activation, and exhibited long-lived transitions to subconductance states at high positive voltages. Complete characterization the gating, ion permeability, and pharmacology of Cx43-G8V, Cx43-A44V, and Cx43-E227D hemichannels will require additional studies.

Cx43 is a widely expressed protein, and is present in the skin across the epidermis^21,57,58^. Most mutations in the *GJA1* gene, encoding Cx43, result in oculodentodigital dysplasia (ODDD)^59^, a disorder that manifests with neuropathies, facial, dental, and digit abnormalities and very rarely skin disease^60^. Recently, *GJA1* mutations were identified in patients with three distinct, non-syndromic, skin-limited diseases, who lacked any of the diagnostic features of ODDD. Cx43-G8V was found in three patients from two unrelated families with PPKCA1^22^, Cx43-A44V was detected in two unrelated patients, one with EKVP and the other with ILVEN^24,27^, and Cx43-E227D was identified in two unrelated patients with EKVP^24^. There is overlap in the clinical features presented in these disorders, suggesting that a common functional consequence, such as augmented hemichannel activity, could underlie the pathology resulting from the three distinct mutations.

As described in the introduction, analysis of mutations in other connexins associated with epidermal disorders has suggested a general role for augmented hemichannel function in the pathology of skin disease^19,20^. Other recent studies have suggested that wild-type Cx43 hemichannel activity was promoted by Cx26 mutations through the formation of heteromeric hemichannels. The first study examined the KID syndrome mutation Cx26-S17F. Although Cx26-S17F was unable to form hemichannels or gap junction channels when expressed alone^61^, it showed significantly increased hemichannel activity when co-expressed with wild-type Cx43^35^. The second showed that two PPK mutations, Cx26-H73R and Cx26-S183F, both failed to form hemichannels when expressed alone in *Xenopus* oocytes. Like Cx26-S17F, co-expression of either Cx26 PPK mutant with Cx43 showed significantly increased hemichannel activity, compared to Cx43 alone. Co-immunoprecipitation showed that Cx43 was efficiently pulled down with either Cx26-H73R and Cx26-S183F, confirming the formation of heteromeric hemichannels^33^. In the present work, we found that expression of skin disease causing Cx43 mutations resulted in augmented membrane currents mediated by active hemichannels. The addition of the data for Cx43, to the cumulative reports on Cx26, Cx30 and Cx31 mutations, makes a strong case that increased hemichannel activity linked to connexin mutations associated with epidermal disorders may contribute to disease pathology.

## Methods

### Molecular cloning

Human Cx43 was cloned into pCS2^+^ ^62^ for functional studies as previously described^33^. Mutant Cx43-G8V, Cx43-A44V, and Cx43-E227D were generated by site directed mutagenesis^63^ using human Cx43 as a template. Cx43-G8V, Cx43-A44V, and Cx43-E227D were cloned into pBlueScript II (Agilent Technologies, Santa Clara, CA) and sequenced prior to being subcloned into pCS2+ for *Xenopus* oocyte expression, or pIRES2-EGFP2 (Clontech Laboratories, Mountain View, CA) for expression in HeLa cells^47^.

### *In vitro* transcription, oocyte microinjection, and pairing

Cx43 constructs in pCS2^+^ were linearized with Not1 and cRNA was transcribed using the SP6 mMessage mMachine (Ambion, Austin, TX). *Xenopus laevis* oocytes were purchased (Xenopus 1, Dexter, MI) and cultured in in modified Barth’s (MB) medium^47^. Oocytes were injected with 10 ng of antisense oligonucleotide against *Xenopus* Cx38^43,44^, followed by connexin transcripts (5 ng/cell). Antisense Cx38 oligonucleotide treated oocytes injected with water, instead of cRNA, served as a negative control.

### Hemichannel current recording

Whole cell hemichannel currents were recorded 24 hours after cRNA injection into *Xenopus* oocytes using a GeneClamp 500 amplifier operated by a PC-compatible computer using a Digidata 1440A interface and pClamp 10 software (Axon Instruments, Foster City, CA). Electrodes (1.5 mm diameter glass, World Precision Instruments, Sarasota, FL) were pulled to a resistance of 1–2 MΩ (Narishige, Tokyo, Japan) and filled with 3 M KCl, 10 mM EGTA, and 10 mM HEPES, pH 7.4. In most cases, cells were recorded in MB medium without added calcium^34^. Hemichannel current-voltage (I–V) curves were obtained by clamping cells at −40 mV and imposing voltage steps in 10 mV increments ranging from −30 to +60 mV. For perfusion experiments testing hemichannel block by cations, extracellular solutions were exchanged using a six channel perfusion valve control system and a slotted bath oocyte recording chamber (VC-6 and RC-1Z, Warner instruments, Hamden, CT).

For patch-clamp recordings of single-hemichannel currents, *Xenopus* oocytes were manually devitellinized in a hypertonic solution consisting of (in mM) 220 Na-aspartate, 10 KCl, 2 MgCl_2_, and 10 HEPES, and then placed in ND96 medium for recovery. Individual oocytes were moved to a recording chamber (RC-28; Warner Instruments) containing the patch pipette solution, which consisted of (in mM): 140 KCl, 1 MgCl_2_, 5 HEPES, 1 CaCl_2_, and 3 EGTA, pH adjusted to 8.0 with KOH. The bath compartment was connected via a 3-M agar bridge to a ground compartment containing the same IPS solution. Single-hemichannel records from voltage steps and ramps were leak subtracted by measuring the leak current during full-closing events and extrapolating linearly with voltage.

### Recording of gap junctional conductance

In *Xenopus* oocyte pairs, junctional conductance (G_j_) was measured by initially clamping both cells in a pair at −40 mV (a transjunctional potential (V_j_) of zero). One cell was subjected to alternating pulses of ±20 mV and the current produced by the change in voltage was recorded in the second cell, which was equal in magnitude to the junctional current (I_j_). Conductance was calculated by dividing I_j_ by the voltage difference, G_j_ = I_j_/(V1 − V2)^64^. Gating properties were determined by recording the junctional current in response to hyperpolarizing or depolarizing V_j_s in 20-mV steps. Steady-state currents (I_jss_) were measured at the end of the voltage pulse. Steady-state conductance (G_jss_) was calculated by dividing I_jss_ by V_j_, normalized to ± 20 mV, and plotted against V_j_.

For recordings of single channel junctional currents, HeLa, or N2A cells were transfected with cDNA corresponding to the mutant connexins. Junctional currents were measured using the dual whole cell patch-clamp technique as described previously^65^. Single gap junction channel currents were visualized during washout of 100% CO_2_ saturated media, which uncouples cells completely. All electrophysiological measurements were obtained using Axopatch 1D patch clamp amplifiers (Molecular Devices, San Jose, CA). Data were acquired by using pClamp 9.2 software; analysis was performed with pClamp 9.2. Currents were filtered at 0.5–1 kHz and sampled at 2–5 kHz.

### Western blotting

Oocytes extracts were prepared as previously described^66^, run on 12% SDS gels and then transferred to nitrocellulose. Western blots were first blocked with 5% milk 0.1% Tween20 in TBS, then probed with polyclonal antibodies against Cx43 (Life Technologies, Carlsbad CA), followed by horseradish peroxidase conjugated secondary antibodies (Jackson Laboratories and GE Healthcare). A monoclonal β-tubulin antibody (Abcam, Cambridge, MA) was used as a loading control.

### Cell transfection

HeLa cells were grown to 50% confluence on 22 mm^2^ coverslips and transfected with wild-type or mutant Cx43 using Lipofectamine 2000 (Invitrogen, Carlsbad, CA) as described^10,47,67^. To facilitate cell survival, the calcium concentration in the culture media was elevated to 4 mM by the addition of supplemental CaCl_2_ 24 hours after transfection.

### Immunofluorescent staining of transfected cells

HeLa cells were fixed in 1% paraformaldehyde in PBS 24–48 hours after transfection and blocked with 5% BSA dissolved in PBS with 0.02% NaN_3_ and 0.1% Tx-100 added. Cells were immunostained with a polyclonal Cx43 antibody followed by a Cy3 conjugated goat anti-rabbit secondary antibody (Jackson ImmunoResearch, West Grove, PA). Cells were photographed on a BX51 microscope using a DP72 digital camera (Olympus America, Waltham, MA).

### Statistical analysis

Differences in data sets were analyzed for statistical significance with Origin 6.1 software (Microcal Software, Northampton, MA). Multiple comparisons were done with one-way ANOVA. The data are presented as the mean ± SEM of the indicated number of experiments. Statistical significance was designated for analyses with p < 0.05.

## Supplementary information

Dataset 1
