# Supplementary material for: Connexin43 mutations linked to skin disease have augmented hemichannel activity
Source: Sci Rep. 2019 Jan 10;9:19. doi: 10.1038/s41598-018-37221-2 (PMC6328547; doi:10.1038/s41598-018-37221-2)

Supplementary Data

Connexin43 mutations linked to skin disease have augmented hemichannel activity

Miduturu Srinivas, Thomas F. Jannace, Anthony G. Cocozzelli, Leping Li[2], Nefeli Slavi, Caterina Sellitto, Thomas W. White,*

Full image blots from Figure 1 to comply with the digital image and integrity policies

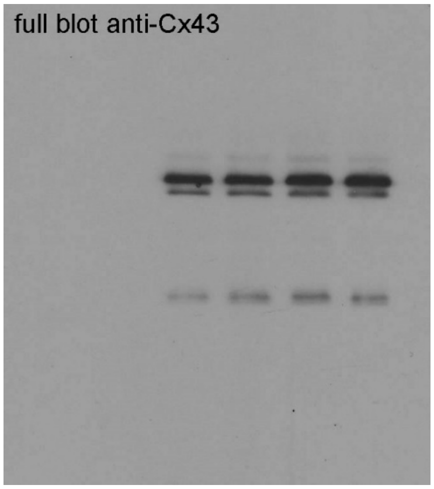

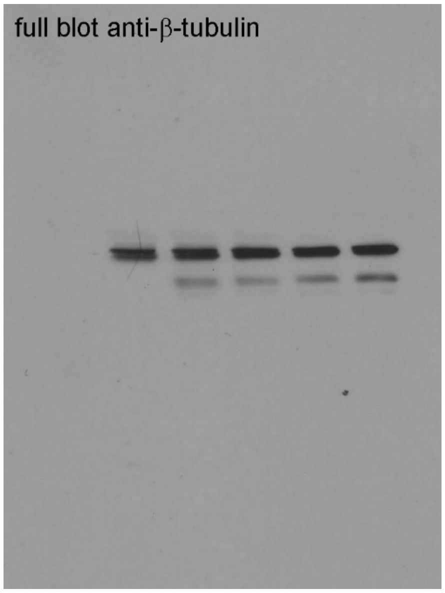

Supplement: Supplementary file 1 — Dataset 1 [file 41598_2018_37221_MOESM1_ESM.pdf]
